# Being blind (or not) to scenarios used in sacrificial dilemmas: the influence of factual and contextual information on moral responses

**DOI:** 10.3389/fpsyg.2024.1477825

**Published:** 2024-10-28

**Authors:** Robin Carron, Emmanuelle Brigaud, Royce Anders, Nathalie Blanc

**Affiliations:** Epsylon EA 4556, Department of Psychology, University Paul Valéry Montpellier 3, Montpellier, France

**Keywords:** sacrificial dilemmas, scenarios, moral responses, moral decision-making, emotions

## Abstract

**Introduction:**

Traditionally, human morality has been largely studied with classical sacrificial dilemmas. A way to advance current understandings of moral judgment and decision-making may involve testing the impact of contexts that are made available to individuals presented with these archetypal dilemmas. This preliminary study focused on assessing whether the availability of factual and contextual information delivered through classical scenarios would change moral responses.

**Method:**

A total of 334 participants were presented with sacrificial dilemmas either with a scenario or without a scenario before performing two moral tasks: one consisted in moral judgment (e.g., *is it acceptable to sacrifice one person to save five?*) and one was related to choice of action (e.g., *would you sacrifice one person to save five?*). In the condition with a scenario, participants were presented with a story describing the dilemma, its protagonists, their roles, the location and some background details of the situation, before answering to the two moral tasks. In the condition without a scenario, participants were only asked to perform the two moral tasks without any additional contextual elements usually provided by the scenario. Participants’ emotions were also measured before and after completing the two moral tasks.

**Results:**

The results indicated that the presence of a scenario did not affect moral judgments. However, the presence of a scenario significantly increased utilitarian action choices (i.e., sacrificing one person in the interest of saving a greater number) and this effect was partially mediated by an increase in the perceived plausibility of the sacrificial action. Regarding emotional reaction to dilemmas, no differences were observed between the two conditions, suggesting that emotions are mainly based on the two moral tasks.

**Discussion:**

These findings underscore the value of carefully considering the role of factual and contextual information provided by the scenarios in moral dilemmas.

## 1 Introduction

A major criticism of studies that investigate human morality through moral dilemma designs is the lack of realism in most classical dilemma sets previously used (Bauman et al., 2014). Originally, these classical dilemma sets came about from philosophers who purposefully designed them for a different framework, that of thought experiments, which were intended to analyze abstract ethical questions. Therefore, the hypothetical and unrealistic nature of these dilemmas was intentional: rather than mirroring real-life situations, their objective was to test ethical principles in truly abstract situations, stripping away real-world elements in order to focus on the validity of principled, philosophical arguments (Foot, 1967; Thomson, 1976). Building on this framework, psychologists interested in studying the mental processes underlying moral judgements have adopted and carried on with these classical dilemmas as a means of studying these mental processes. Nevertheless, these fundamental studies using classical dilemmas led to significant discoveries and the development of major theories that are still recognized today, such as dual process theory (Greene et al., 2001, 2004), which establishes that both intuitive and deliberative processes play an important role in human moral decision-making. However, while these dilemmas served to establish such foundational insights into the psychology of human morality, and offer a simplified, canonical paradigm to its investigation, an increasingly influential critique has emerged in the scientific community, namely that these dilemmas are overly lacking in external validity. In response, many researchers now argue for using more realistic moral dilemmas that more closely correspond to how people make decisions in everyday life.

Different strategies have been proposed by researchers to reduce this gap between abstract dilemmas and lived experiences. For instance, Carron et al. (2022) identify two lines of research that have attempted to overcome this lack of realism: one proposes to increase perceived realism through immersive virtual universes (e.g., simulations, virtual reality), while the other through anchoring dilemmas in more relatable, everyday, or recent, real-life events. For example, in the latter, researchers have incorporated sacrificial dilemmas in peace or war contexts (e.g., Watkins and Laham, 2019), military or professional contexts (Christen et al., 2021), autonomous and non-autonomous vehicles contexts (e.g., Bonnefon et al., 2016; Bruno et al., 2022, 2023, 2024), or health crisis contexts such as the recent COVID-19 crisis (e.g., Carron et al., 2022; Kneer and Hannikainen, 2022). Historically-grounded facts have also been used to increase realism (Körner and Deutsch, 2023), in which clues such as the place and date of events were found to increase the perceived realism of the dilemmas.

A unifying adaptation that can be recognized in these researches that aimed to render such dilemmas less abstract and overcome their previously poor degree of external validity is the addition of *context*, whether through immersive virtual reality or more immersive and relatable details. Therefore, researchers have qualified the classical dilemma sets as too sparsely contextualized (e.g., Kusev et al., 2016; Schein, 2020; Christen et al., 2021; Carron et al., 2022) or, overly simplistic and missing key contextual factors. For example, classical dilemmas such as the famous trolley dilemma (Foot, 1967), provide very little information compared to what a human being can typically and readily perceive in a real-life dilemma, information that is usually crucial for arriving at a satisfiable moral response. Therefore, several works can be noted, as herein, where researchers retained the framework of classical (e.g., sacrificial) moral dilemmas, such as the famous trolley dilemma, and investigated the introduction of additional contextual information at different degrees. Kusev et al. (2016) showed that in the trolley or bridge dilemma, providing complete information about the sacrificial action and its consequences increased the proportion of utilitarian choices and reduced response times, compared to scenarios that provided only partial information. Consistent with this work, Körner et al. (2019); see also (Carron et al., 2022) observed an increase in utilitarian responses when providing information that presented the solution action as plausible. This work builds upon this line of research by examining more broadly the reach of such contextualization: to what lengths may it affect the variables of judgment of what is socially acceptable, one’s personal choice, the perceived realism of the dilemma, and the plausibility of the consequences of the choice, and is there a mediational trend? To our knowledge, no previous work has yet examined the relationship between these four variables and contextualization.

The previously mentioned dual-process theory (Greene, 2007; Greene et al., 2001, 2004, 2008), albeit derived from classical dilemmas, remains a fundamental framework for addressing these questions, as it is capable of generating several hypotheses in how contextual information may in fact impact moral experience and responses. According to this theory, two distinct systems are involved in moral judgments: (1) an automatic emotional process based on intuition and affect (rapid and largely unconscious), and (2) the controlled cognitive process corresponding to conscious reasoning (slow and laborious). Moral judgments can be understood as stemming from either conflict or cooperation between these two systems. Conflict is more often expected in dilemmas that would elicit strong emotions, such those that are personally charged, or involve directly interacting (especially physically) with other beings and influencing their outcomes. A good example is the “footbridge dilemma,” where the respondent must decide whether he/she would push an individual off a bridge to stop a trolley and thus save five other people. It has been found that this type of dilemma tends to strongly activate System 1, linked to rapid emotional reactions. Acting as a “moral alarm,” System 1 dominates, preventing the slower, more deliberate reasoning of System 2 from engaging. As a result, this conflict increases the likelihood that individuals make a deontological judgment: that is, avoiding intentionally harming or sacrifice of others, even if it could save more lives (see also Capraro, 2024, in highly emotional contexts, individuals are found more intuitively and automatically inclined towards self-preservation and aversion to harm). Conversely, cooperation between the two systems occurs in dilemmas that are less emotionally charged, such as impersonal dilemmas (i.e., where there is no direct or immediate contact with another person). Take, for instance, the “trolley dilemma,” where instead of potentially pushing a person, a lever may be pulled to divert a trolley onto another track, which would kill one person but save five others. This dilemma is found to less strongly activate System 1, linked to a more subdued emotional response, allowing System 2 to engage. System 2 is then more likely to evaluate the consequences of the two alternatives (Cushman et al., 2006), in which the decision to sacrifice one life to save several others becomes morally more acceptable, known as a utilitarian judgment. Thus, this framework of cooperation between the two systems, or at least for System 2 to not be too prematurely overridden by System 1, has been identified to increase the likelihood of utilitarian judgments.

Based on dual-process theory (Greene, 2007; Greene et al., 2001, 2004, 2008), we therefore hypothesize that when there is a greater availability or richness of factual and contextual information to be processed by individuals, System 2, or a greater cognitive appraisal of a dilemma will follow, increasing the likelihood of utilitarian responses. Factual and contextual information may be defined as descriptive, factual details that do not alter the fundamental meaning of the story but help situate the possible actions that one has to choose from. These details allow individuals to better understand the stakes and consequences of the situation without introducing interpretative or narrative bias. Initial findings (e.g., Kusev et al., 2016) have suggested that considering these nuances promotes the inhibition of System 1, which relies on rapid, instinctive emotional reactions, and encourages a deliberative process associated with System 2. In other words, processing contextual information activates cognitive mechanisms (Moss and Schunn, 2015) that enable a more reflective evaluation of actions and their consequences. Thus, integrating factual and contextual information into moral decision-making leads individuals to favor judgments based on consequences, thereby increasing the likelihood of a utilitarian response. However conversely, it is equally important to recognize the possibility that certain types of contextual information may instead lead to more deontological choices. In that case, some studies provided contextual information which was not factual but personalized, such as proximity (Tassy et al., 2013), age (Kawai et al., 2014), or gender (FeldmanHall et al., 2016) of the person being sacrificed. The present study specifically examines the contribution of non-personalized factual information provided in classical dilemma scenarios to test whether the moral responses produced are sensitive to the presence or absence of this type of factual information.

It is also important to make the distinction that morality literature has nuanced that there is an important difference between choice of action and judgment in moral responses (Tassy et al., 2013), which is taken into account in the present investigation. That is, assessing whether contextual information only affects choice of action (towards utilitarianism), or choice as well as judgment, as it is established that these two are not necessarily predictive of one another. For example, one may judge that it is acceptable to sacrifice one person in order to save 5 others, without committing to actually realizing that sacrifice. Works have established that these two types of internal decisions, what is right and what will I do, are often based on highly normative principles (e.g., forbidden to kill), common sense (Tassy et al., 2013), but also different cognitive processes (Tassy et al., 2012). Our consideration of recent works suggests that these cognitive processes take into account contextual information and primarily influence moral action rather than judgment. For example, Tassy et al. (2013) observed that providing participants with information about the person to be sacrificed (a family member vs. a stranger) influenced their choice of action more than their judgment. Similarly, Carron et al. (2022) showed that information about the plausibility of the resolution action in COVID-19 dilemmas also influenced participants’ moral actions rather than their judgments.

The Consequences, Norms, and generalized Inaction (CNI) model (Gawronski et al., 2017) provides a theoretical framework to explain the observed differences between these two types of moral responses: action (or inaction) and judgment. In this model, the authors propose to quantify participants’ sensitivity to the consequences of the action (parameter C), to moral norms (parameter N), as well as their preference for action or inaction (parameter I), in order to identify the processes underlying moral responses. According to the CNI model (Gawronski et al., 2017), these processes are sequential and lead individuals to successively consider the consequences of the action (parameter C), then the applicable norms (parameter N) and, in the absence of this information, their preferences for action or inaction. Thus, Gawronski et al. (2017) tested the sensitivity of participants to different parameters depending on whether they had to make a moral judgment (e.g., is it acceptable to kill?) or a choice of action (i.e., “would you kill?”). The results showed that those who had to make a choice had a stronger preference for inaction than those who had to make a judgment, but that the former were also less sensitive to social norms than the latter (see Gawronski et al., 2020 for similar results). The weight of norms would therefore be less important for decision making than for moral judgment. Therefore, these works are also coherent with our hypothesis that contextual information provided in moral scenarios should influence actions more than judgments.

The present study aimed to test this hypothesis by comparing participants’ responses in a between-subjects design. Traditional sacrificial dilemmas were presented under two distinct conditions. In one condition, a scenario provided factual and contextual information about the moral dilemma, and in the other one, no such information was provided to contextualize the dilemmas. In both conditions, participants were asked to provide two types of moral responses: one focused on moral judgment and the other on choice of action. We expected that providing factual and contextual information through the scenario would increase the degree of utilitarian responses, the perceived realism of the dilemma, and the perceived plausibility of the action choices and their consequences. More specifically, we hypothesized that the link between the presence of the scenario and increased utilitarian responses may be partially mediated by perceived realism and/or perceived plausibility of the action. In other words, the presence of scenario was expected to indirectly promote utilitarian responses, suggesting that participants would be more inclined to choose actions that maximize overall well-being when the situation felt more realistic and the actions seemed more plausible.

As a secondary endeavor, this study sought to examine the link between contextual information provided in the sacrificial dilemma scenarios and emotional experience of participants presented with these moral dilemmas. Several studies have explored the emotional reaction during sacrificial dilemmas (e.g., Choe and Min, 2011; Szekely and Miu, 2015) and showed that participants mainly feel sadness, anger, disgust, guilt, shame and empathy. Further results from Horne and Powell (2016) show that some of these emotions (especially anger and disgust) are involved in moral responses. According to Moll and de Oliveira-Souza (2007; see also Tassy et al., 2012), the wide range of emotions felt when people are confronted with a situation involving physical harm could be categorized as “self-focused” or “other-focused” emotions. The former are associated with imagining oneself in a moral dilemma with the perspective of personally harming another person (e.g., anger, disgust, and sadness) and the latter are associated with imagining the consequences of the harming action (e.g., guilt, shame). Although the role of emotions in moral judgment is a topic of much debate in psychology (see Cameron et al., 2015; Landy and Goodwin, 2015; Donner et al., 2023 for meta-analyses), our study aimed to also determine whether the presence of a scenario can elicit stronger emotional reactions to sacrificial dilemmas, by which participants filled out validated emotion scales in each moral experimental condition.

## 2 Methods

### 2.1 Participants

To ensure adequate statistical power for this study, power analyses were conducted *a priori* using G*Power statistical software (Faul and Erdfelder, 1992). Necessary sample size was computed for the analyses herein, namely two-way repeated-measures ANOVAs that take into account a within-between interaction, in which a small effect size of 0.10 was assumed for all effects. This conservative parameterization ensures that if the appropriate number of participants as suggested by the analysis is obtained, the study would have sufficient power to detect statistically significant effects, even if only small effect sizes were present. With a significance level of *α* = 0.05 and a power of (1-*β*) = 0.95, the analysis indicated that a sample size of 328 participants was required. In line with this result, 334 individuals participated in the experiment. These participants were all undergraduate students (287 female, *M*_age_ = 20.18, *SD* = 2.75), native French speakers, and first-year students in psychology from the University of Montpellier 3 (France). They participated between September 19 and December 02, 2022. Informed consent was obtained from all students prior to participating in any of the tasks. They were informed that their responses remained anonymous in respect of the Data Protection law. All students received course credit as compensation.

### 2.2 Materials

#### 2.2.1 Moral dilemmas

Three comparable high conflict personal dilemmas (Euthanasia dilemma, Vitamins dilemma,^1^ and Footbridge dilemma) were selected from a previously used classical moral dilemma set (see Greene et al., 2001, 2004). These dilemmas describe a variety of situations all the while being tightly comparable along the following defining characteristics. Namely, each dilemma involved killing one person in order to save five others and the potential victims were unknown to the participants; the participant was presented as the main protagonist of the situation (i.e., the one who was supposed to carry out the moral violation), the sacrifice that the protagonist had to make involved physical contact or the use of personal force (e.g., such dilemmas have traditionally been labeled “personal harm”); the consequences of the action were only for the benefit of others, never for the benefit of the protagonist himself (“Other-Beneficial dilemmas”).

The three classical dilemmas previously mentioned were presented either with a scenario or without a scenario. The “With Scenario” condition corresponded to the classic experimental paradigm in moral psychology, since a short scenario provided factual and contextual information related to the dilemmas. Specifically, for each of the three dilemmas, the scenario described the scene and its protagonists, the resolution action, and the consequences of this action. The sentence introducing the resolution action, as well as the one presenting the consequences, were always the same: the first insists on the fact that there is only one way to save the 5 people, and the second underlines the consequences for each of the protagonists (saved or killed, see Table 1 for the Footbridge dilemma scenario). In the “Without Scenario” condition, participants discovered the dilemmas only through two moral questions (see Table 2 for Footbridge dilemma).

**Table 1:** Footbridge dilemma in the condition with a scenario.

A runaway trolley is heading down the tracks toward five workmen who will be killed if the trolley proceeds on its present course. You are on a footbridge over the tracks, in between the approaching trolley and the five workmen. Next to you on this footbridge is a stranger who happens to be very large. The only way to save the lives of the five workmen is to push this stranger off the bridge and onto the tracks below where his large body will stop the trolley. If you do this, the stranger will die, but the five workmen are saved.

**Table 2:** The two moral questions associated with Footbridge dilemma.

Moral judgment	Choice of action
How appropriate is it for you to push a stranger off the bridge and onto the tracks below, in order to use his large body to stop a runaway trolley and save five workmen on the tracks?	Would you push a stranger off the bridge and onto the tracks below, in order to use his large body to stop a runaway trolley and save five workmen on the tracks?

#### 2.2.2 Moral responses measures

As Tassy et al. (2013) have shown, there is often a significant discrepancy between what an individual considers morally acceptable (i.e., an abstract moral *judgment*) and their hypothetical or desired behavior in moral dilemmas (i.e., a choice of *action*). To capture this, participants consecutively answered two questions, one focused on moral judgment and the other on choice of action (see Table 1 for the Footbridge dilemma).

For the moral judgment task, participants rated the extent to which the utilitarian action was appropriate or not. All questions were framed in the following manner: “How appropriate is it for you to X [e.g., ‘push a stranger off the bridge …’]?”

For the choice of action task, participants were asked whether they would perform the utilitarian action (i.e., choice of action). All questions were framed in the following manner: “Would you X [e.g., ‘push a stranger off the footbridge …’]?”

These two questions were answered on a 6-point scale (1 = not at all; 6 = definitely) with higher scores being closer to utilitarian responses. The 6-point Likert scale, as opposed to binary responses, was chosen to capture more nuances in moral responses. To reduce potential central tendency bias, an even-numbered scale was used, which encouraged participants to take a clear stance as neutral values were impossible.

#### 2.2.3 Perceived realism measures

In line with authors who argue that realism perceptions possess multiple dimensions which are important to assess (e.g., Busselle and Bilandzic, 2008; Carron et al., 2022; Hall, 2003, 2017), we measured the following three sub-dimensions: perceived plausibility, typicality, and factuality. The question related to plausibility was: “How probable do you think it is that this dilemma could possibly happen in real life?”; typicality: “How probable do you think it is that this dilemma reflects people’s past and present experiences?”; and factuality: “How probable do you think it is that this dilemma depicts something that really happened?.” Responses to these three perceived realism measures were rated on a 6-point scale (1 = not at all, to 6 = definitely).

#### 2.2.4 Plausibility of action measures

Following the method of Körner et al. (2019, see also Carron et al., 2022), for each dilemma, participants rated the plausibility of the sacrificial actions presented by answering the two following questions: “How probable do you think it is that this action would save the five people?,” “How plausible is it that there are no better alternative actions–no reasonable actions to save the five people?.” Responses were rated on a 6-point scale (1 = not at all, to 6 = definitely).

#### 2.2.5 Emotional scales

Participants’ emotional states were assessed using self-report survey scales of discrete emotions. In line with previous works, we considered here the traditional emotions assessed in classic sacrificial dilemma paradigms, namely: sadness, anger, disgust, guilt, shame, and empathy (Choe and Min, 2011; Szekely and Miu, 2015). Participants were asked to rate the intensity with which they felt each of these 6 emotions at the time of measurement (i.e., at the beginning of the experiment and after providing the moral responses) using a continuous slider ranging from 0 (indicating very low intensity) to 20 (indicating very high intensity). Slider format responses have been found to show high validity and reliability, especially for repeated-measures experimental designs (Imbault et al., 2018).

### 2.3 Procedure

Participants were tested using a questionnaire programmed on Qualtrics.^2^ This research was approved by the Institutional Review Board (IRB) of Paul Valéry University with the protocol number IRB00013686-2023-07-CER, and written consent was obtained from all participants.

Moral dilemmas were presented either with a scenario or without a scenario. Therefore, after providing informed consent and reading the instructions, 334 participants were randomly assigned to one of the two conditions (*n* = 167 per condition). The experiment was conducted in a quiet room, with participants seated individually at computers equipped with the Qualtrics software, in groups of 30 to 40 subjects. Strict silence was maintained in the room to ensure the absence of distractions. In the first part of the questionnaire, they were asked to answer questions concerning their emotional state at the time of measurement. All emotional evaluation questions were presented on a single screen.

Immediately after, participants responded to the moral dilemmas with scenario or without scenario, depending on their random group assignment. All participants responded to the three dilemmas (i.e., Euthanasia dilemma, Vitamins dilemma, and Footbridge dilemma) in a random order.

Participants in the condition with scenario, first received description of the dilemma describing its protagonists, their roles, the location and some background details of the situation (see Table 1). They could study the details on this screen as long as they preferred (no questions were available on this screen) before using their mouse to move to the next screen. After reading the scenario, participants were presented with a subsequent screen containing the moral tasks via two consecutive questions related to that dilemma: the first focused on moral judgment, and the second on the choice of action (see Tassy et al., 2013, for a similar procedure). This procedure was repeated for the other two dilemmas.

In the condition without scenario, participants did not read any scenarios. Instead, they directly answered the two moral questions for each of the three dilemmas. For each dilemma, both moral questions were presented on the same screen. This procedure was repeated for the other two dilemmas. All text was provided in black font (Arial, size 12) in blocks of text on a white background.

The moral tasks were briefly introduced by stating that they refer to serious situations that could be seen as unpleasant but require making a difficult choice. Participants were asked to be as honest as possible in their responses, knowing that there was no right or wrong answer. After giving their moral answers, participants rated their emotional state again. Therefore, emotional state was assessed twice per condition: at the beginning of the experiment and after providing the moral responses (see Choe and Min, 2011 for a similar procedure).

In the second part of the questionnaire, the participants rated the realism of each of the 3 dilemmas and the plausibility of sacrificial action presented in each of the 3 dilemmas. To make these assessments, they were shown the 3 dilemmas again, with or without scenario, depending on the experimental condition. Finally, participants provided demographic information (i.e., age, gender). For the two conditions, participants were given unlimited time to complete the survey.

**Figure 1:**
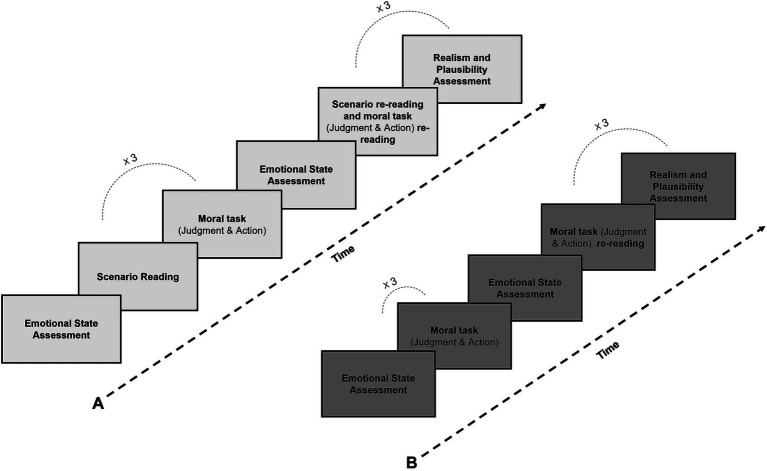
Experimental procedure in with scenario (A) and without scenario (B) conditions.

The experimental procedure is represented in Figure 1.

## 3 Results

### 3.1 Data analysis

**Figure 2:**
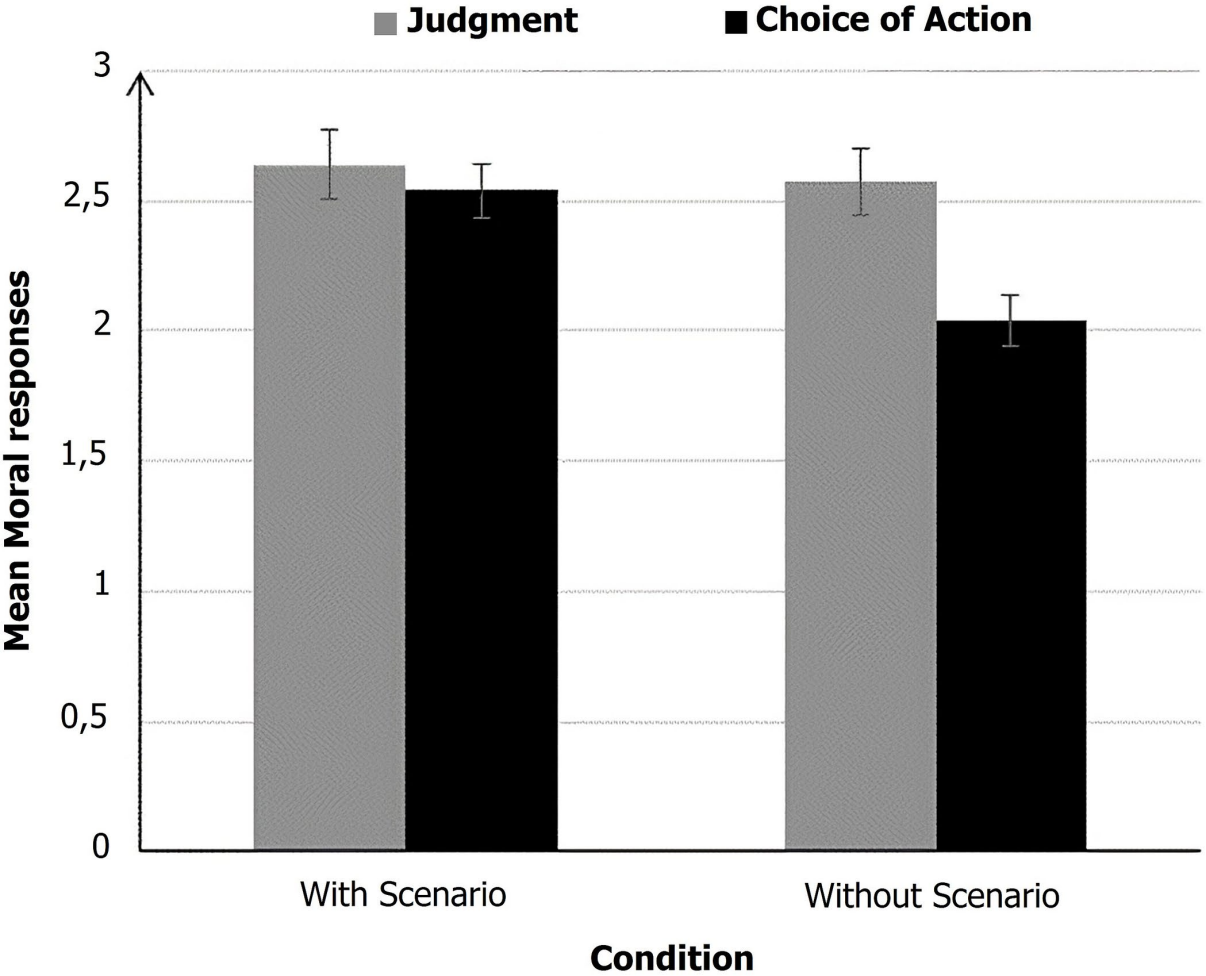
Mean moral responses as a function of condition (with scenario vs. without scenario) and type of moral response (judgment vs. choice of action). higher scores (max = 6) are closer to utilitarian responses.

The data were analyzed using Jamovi^®^ software (The jamovi project, Sydney, Australia; Version 2.6.13; https://www.jamovi.org/). Three different mixed linear models were calculated, with Moral Responses as the dependent variable in each model. The first model included Type of moral response (Judgment vs. Choice of action) and Scenario (With vs. Without) as fixed effects, with Dilemma and Participant as random factors. The second model included Realism (Factuality vs. Plausibility vs. Typicality) and Scenario (With vs. Without) as fixed effects, with Dilemma and Participant as random factors. The third model included Plausibility (Consequences vs. Alternative) and Scenario (With vs. Without) as fixed effects, with Dilemma and Participant as random factors. The models were estimated using the gamlj_mixed function from the GAMLj3 package (Gallucci, 2019; https://github.com/gamlj/gamlj). Tukey’s pairwise comparisons were conducted for *post hoc* tests. The level of significance was set at *p* < 0.05. We used a Linear Mixed Model (LMM) analysis with restricted maximum likelihood (REML) estimation, and degrees of freedom were calculated using the Satterthwaite method, as implemented in the General Analyses for Linear Models module of Jamovi. For the Direction of change analysis, we used Chi-squared tests to assess the differences in the proportions of participants shifting towards more or less utilitarian choices across conditions. For the Emotional State analysis, we conducted a series of 2 × 2 ANOVAs to compare emotional reactions before and after the task across conditions, and Pearson correlations were performed to examine the relationship between emotional changes and moral responses. Additionally, a mediation analysis was run to test the indirect effects of scenario presentation on choice of action through perceived plausibility. All tests were carried out with a significance threshold set at *p* < 0.05. This interaction indicated that participants were more utilitarian in the condition with scenario than in the condition without, in their choice of action” on line 629. Here is the modified sentence: “This interaction indicated that participants were more utilitarian in the condition with scenario than in the condition without, in their choice of action (see Figure 2).

### 3.2 Moral responses

The linear mixed model analysis revealed several significant effects (see Table 3). Responses to Choice of action were significantly less utilitarian (*M* = 2.29, *SD* = 0.98) than responses to Judgment (*M* = 2.61, *SD* = 0.94). Scenario was also significant, showing that moral responses were less utilitarian in the condition without scenario (*M* = 2.31, *SD* = 0.85) than in the condition with scenario (*M* = 2.59, *SD* = 0.86). Additionally, a significant interaction between Scenario and Type of moral response was found. This interaction indicated that participants were more utilitarian in the condition with scenario than in the condition without, in their choice of action. Indeed, a *post hoc* analysis (Tukey test) revealed that participants were less inclined to choose the utilitarian action in the absence of the scenario than in its presence (*p* < 0.001). However, their moral judgment was the same in both conditions (with or without scenario), *p* = 0.93.

**Table 3:** Linear mixed-effects model results for moral responses.

Fixed effects	Estimate	*SE*	t	*p*-value
Intercept	2.26	0.54	4.15	0.03
Type of moral response	0.44	0.06	7.49	< 0.001
Scenario	−0.29	0.09	−3.06	0.002
Type of moral response * Scenario	0.43	0.09	4.57	0.002

Random effects	SD
Dilemma (intercept)	1.08
Participant (intercept)	0.74
Residual	1.06
Marginal R^2^	0.03
Conditional R^2^	0.61
AIC	6,414

However, judgments and choices of action do not provide full information on the way the presence of a scenario influences moral responses. Indeed, we can gain further insight into this influence by analyzing the direction of change between the two moral responses. We can examine how a given person changed (or did not change) his/her response between the initial judgment and the final action choice. More specifically, people can make a deontological judgment and choose a utilitarian action or, conversely, make a utilitarian judgment and choose a deontological action, or not change their response. It should be noted that this analysis of the direction of change in responses to dilemmas is directly inspired by Bago and De Neys (2017, 2019). Based on the dual-process hypothesis, we can predict that people in the condition with scenario, because they have more factual and contextual information, will be proportionally more likely to switch from a deontological judgment to a utilitarian choice than those without a scenario.

For the analysis aimed at tracking the direction of change in responses (Judgment and Choice of action) between conditions, we calculated the difference between the second moral response (i.e., the choice of action) and the first moral response (i.e., the judgment). For each participant, we calculated the difference between the mean score for the choice of action responses and the mean score for the judgment responses. A positive score indicates that the participant has moved towards a more utilitarian choice of action than their initial judgment, and a negative score indicates that the participant has moved towards a less utilitarian choice of action. A zero score corresponds to no change between the two moral responses.

Chi-squared tests revealed that participants who moved towards a more utilitarian choice of action than their initial judgment were proportionally more numerous in the condition with a scenario (31.7%, *N* = 53) than in the condition without scenario (7.8%, *N* = 13), χ^2^ (1) = 30.21, *p* < 0.001. On the other hand, participants who moved towards a less utilitarian choice of action than their initial judgment were proportionally more numerous in the condition without scenario (68.3%, *N* = 114) than in the condition with a scenario (49.7%, *N* = 83), χ^2^ (1) = 11.89, *p* < 0.001. The proportion of participants who did not change their position between the two moral responses was identical in both conditions (23.9%, *N* = 40 in condition without scenario; 18.6%, *N* = 31 in condition with a scenario), χ^2^ (1) = 1.45, *p* = 0.23.

### 3.3 Perceived realism

The linear mixed model analysis for realism revealed significant effects (see Table 4). Responses to the question about plausibility were significantly lower (*M* = 3.56, *SD* = 1.80). than responses to the question about factuality (*M* = 4.15, *SD* = 1.71). The Scenario did not show a significant effect on realism.

**Table 4:** Linear mixed-effects model results for perceived realism.

Fixed effects	Estimate	*SE*	t	*p*-value
Intercept	3.97	0.65	6.15	0.03
Realism	−0.60	0.05	−11.17	< 0.001
Scenario	−0.14	0.10	−1.41	0.16

Random effects	SD
Dilemma (intercept)	1.12
Participant (intercept)	0.83
Residual	1.19
Marginal R^2^	0.03
Conditional R^2^	0.59
AIC	10,187

### 3.4 Perceived plausibility of the sacrificial action

The analysis of plausibility also revealed significant effects (see Table 5). Participants rating scenarios as more plausible in the With scenario condition (*M* = 3.31, *SD* = 1.66) compared to the Without scenario condition (*M* = 3.03, *SD* = 1.70). Responses to the question about the alternative being significantly lower (*M* = 2.57, *SD* = 1.55) than those about the consequences (*M* = 3.78, *SD* = 1.60).

**Table 5:** Linear mixed-effects model results for plausibility of the sacrificial action.

Fixed effects	Estimate	SE	t	*p*-value
Intercept	3.17	0.08	41.59	< 0.001
Plausibility	−1.20	0.06	−21.06	< 0.001
Scenario	−0.28	0.11	−2.42	0.02

Random effects	SD
Dilemma (intercept)	0.09
Participant (intercept)	0.90
Residual	1.28
Marginal R^2^	0.13
Conditional R^2^	0.43
AIC	7,151

### 3.5 Relationship between scenario (with vs. without), perceived plausibility of the sacrificial action and choice of action

Following the mediation modeling method proposed by Baron and Kenny (1986), a mediation analysis was carried out to test whether the relationship between the presence of a scenario and Choice of action is mediated by the Plausibility of the sacrificial action. In this method for testing the mediation hypothesis, there are two paths to the dependent variable. The independent variable (Scenario, With coded as 1 vs. Without coded as 0) must predict the dependent variable (Choice of action), and the independent variable must predict the mediator (Plausibility of the sacrificial action). Mediation is tested through three regressions: (1) Independent variable predicting the dependent variable; (2) Independent variable predicting the mediator; and (3) Independent variable and mediator predicting the dependent variable. In order to test the significance of indirect effects (i.e., mediation) of Scenario on Choice of action through Plausibility of the sacrificial action, we used the bootstrapping procedure for a sample of 1,000 and a confidence interval (CI) of 95%. Indirect effects were considered significant if the bootstrapping confidence interval did not include zero (Frazier et al., 2004). Also, in order to simplify the modeling, the mean of the two plausibility measures (correlated by *r* = 0.58) described in the previous subsection was modeled instead of the two measures separately.

**Figure 3:**
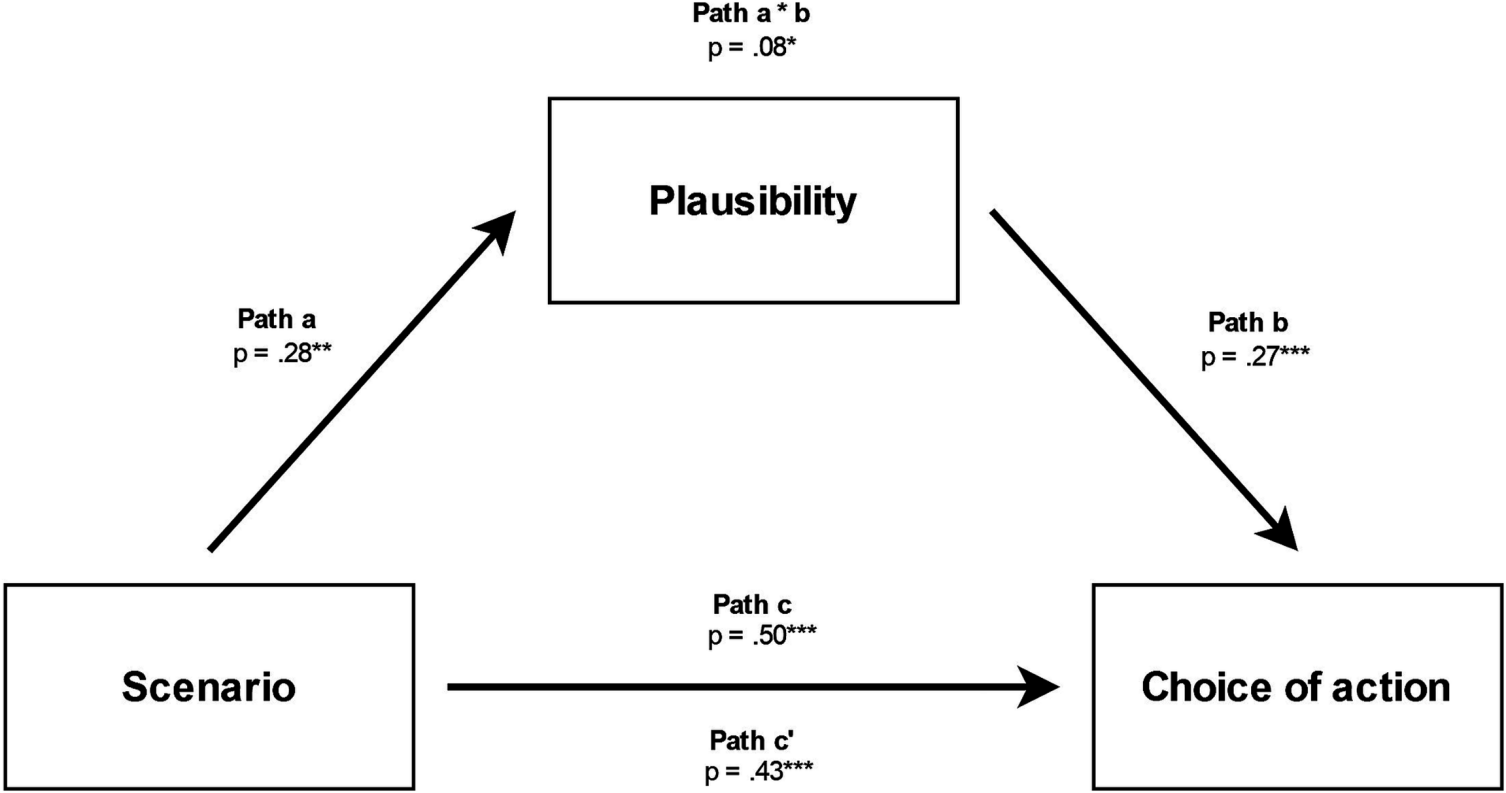
Relationships between scenario, plausibility of sacrificial action and choice of action * *p* < 0.05, ** *p* < 0.01, *** *p* < 0.001.

As shown in Figure 3, the presence of a Scenario was shown to significantly predict Plausibility (*a* = 0.28, *p* < 0.01), Plausibility to predict Choice of Action (*b* = 0.27, *p* < 0.001), and this indirect path with Plausibility to mediate the link between Scenario and Choice of Action to be significant (a*b = 0.08, *p* = 0.03), as the bootstrapping confidence interval did not include zero (see Table 6). The effect of Scenario on Choice of action remained significant after controlling for Plausibility, this suggests that Plausibility plays a role of partial mediation.

**Table 6:** Summary of the mediation analysis.

	Effect	Estimate	*SE*	95% CI
Indirect effect	Scenario ➔ Plausibility ➔ Choice of action	0.08	0.03	[0.02, 0.15]
Component	Scenario ➔ Plausibility	0.28	0.11	[0.05, 0.50]
	Plausibility ➔ Choice of action	0.27	0.05	[0.18, 0.37]
Direct effect	Scenario ➔ Choice of action	0.43	0.10	[0.23, 0.62]
Total effect	Scenario ➔ Choice of action	0.50	0.10	[0.29, 0.71]

### 3.6 Emotional states

**Figure 4:**
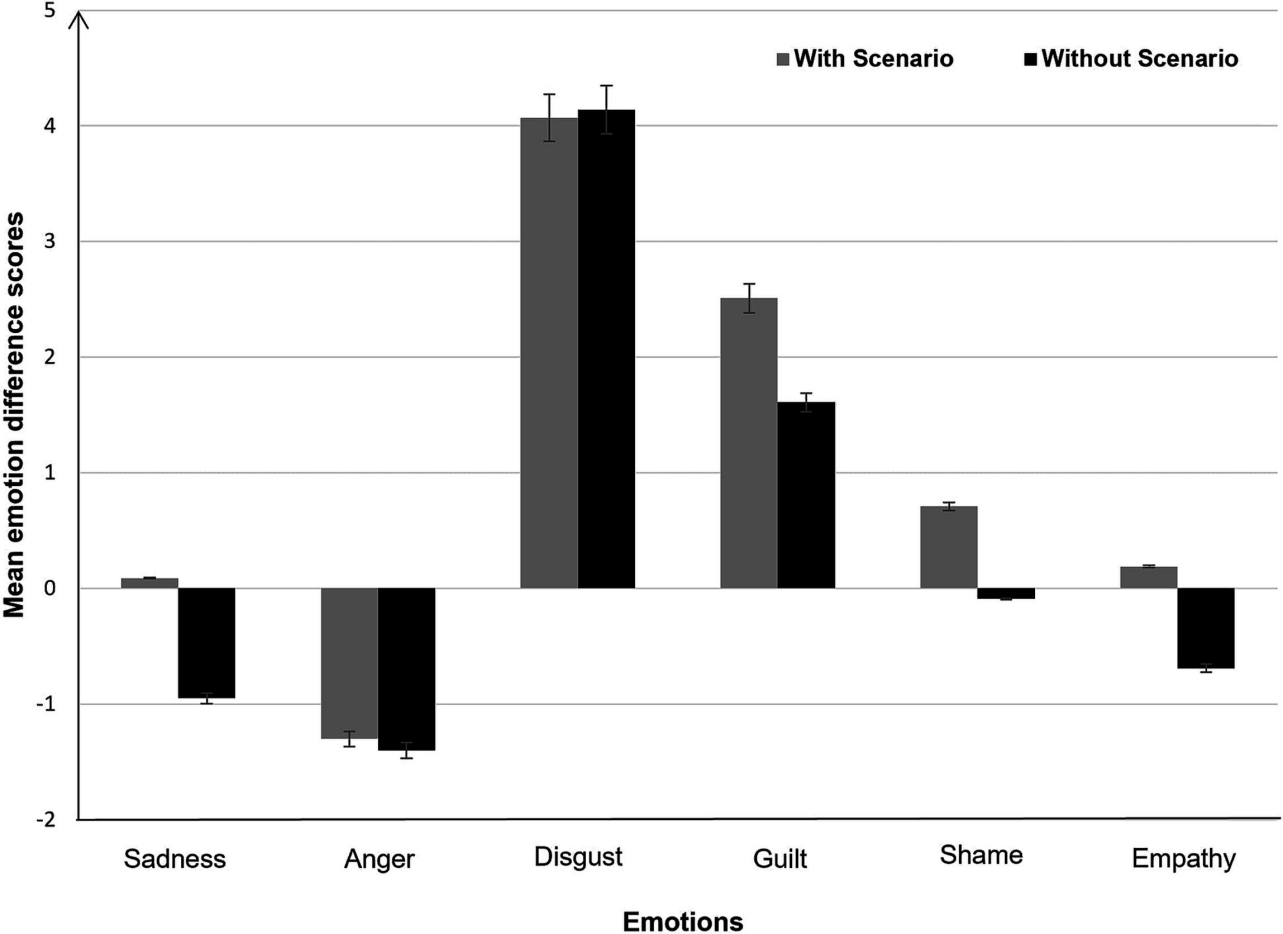
Mean emotion difference scores as a function of scenario (with vs. without). Error bars represent ±1 standard error.

To examine whether the emotional state of participants faced with moral tasks fluctuated according to the presence or absence of a scenario, we computed an emotional reaction score. For each of the 6 emotions, we calculated an emotion reaction score by subtracting participants’ pre-test emotion ratings from their post-test emotion ratings (for similar data processing, see Horne and Powell, 2016). Mean emotional reaction scores for each condition are shown in Figure 4.

We conducted a series of 2 × 2 ANOVAs, one for each of the 6 emotions. The Scenario (With vs. Without) was the between-participant factor, and Emotional Reaction (pre-test vs. post-test) the within-participant factor. We observed a significant main effect of Emotional Reaction factor for three emotions: guilt, disgust and anger. Participants reported feeling more guilt after providing moral responses than at the beginning of the experiment, *F* (1, 332) = 27.88, *p* < 0.001, η^2^_p_ = 0.08. They also reported feeling more disgust after providing moral responses than at the beginning of the experiment, *F* (1, 332) = 134.68, *p* < 0.001, η^2^_p_ = 0.29. On the other hand, participants reported feeling less anger after providing moral responses than at the beginning of the experiment, *F* (1, 332) = 15.40, *p* < 0.001, η^2^_p_ = 0.04. No other effects were significant. These results indicate that emotional reactions were the same whether the scenario was present or absent.

We also examined the relationship between participants’ emotional reactions and their moral responses. We correlated participants’ difference score for each emotion scale and their moral responses (i.e., Judgment and Choice of action). We observed small but reliable correlations: the first, between participants’ moral judgment and the change in their ratings of disgust (*r* = 0.14, *p* = 0.01) and, the second, between participants’ choice of action and the change in their ratings of anger (*r* = 0.13, *p* = 0.02).

## 4 Discussion

The main aim of this study was to investigate how the presence of a scenario that provides factual and contextual information about a sacrificial dilemma may influence one’s moral responses to it. To this end, we compared the judgments and choices of action that individuals expressed when faced with classic sacrificial dilemmas, presented either with a scenario or without a scenario. As expected, the presence of a scenario did not affect their moral judgments (i.e., is it acceptable?) but significantly increased their choices of action to be more utilitarian (i.e., sacrificing a person in the interest of saving a greater number). This result can be explained by the understanding that the factual and contextual information provided by the scenarios primed the fact that the sacrificial action is not only the only possible alternative (i.e., ‘the only way to save the lives of the five is to …’) but also a profitable action (i.e., ‘if you do this, the five lives will be saved’). The crucial presence of these two pieces of information (i.e., the plausibility of alternatives and the plausibility of the stated consequences) within the scenario led to the sacrificial action being perceived as more plausible.

This result supports the findings of several authors who had already highlighted the influence of the plausibility of sacrificial actions on moral responses (e.g., Carron et al., 2022; Körner et al., 2019; Kusev et al., 2016). Like Carron et al. (2022), we showed that participants were more inclined to choose the utilitarian action if the stated consequences seemed certain and if there was no other way to save the lives of the five people concerned. Note that the plausibility of sacrificial action does not predict moral judgment but only choice of action (see Carron et al., 2022 for similar results). According to Tassy et al. (2012), moral judgment and moral choice may be underlined by distinct psychological mechanisms. The psychological mechanism involved in the judgment would be a more impersonal assessment, distanced from the situation and mainly influenced by moral rules (‘it’s forbidden to kill’, ‘do no harm’). In line with this explanation, Gawronski et al. (2017) showed a greater sensitivity to moral rules in participants producing a moral judgment compared to those producing a choice of action. Factual and contextual information, particularly that relating to the plausibility of the action, therefore has less influence on judgment than on the choice of action. Tracking the direction of change in responses (i.e., between the initial judgment and the choice of action) in each of the two conditions also gave more information on the influence of the scenario. Our results showed that the proportion of participants who moved towards a more utilitarian choice of action than their initial judgment was higher in the condition with scenario than in the one without scenario, which also demonstrates the influence of more factual and contextual information on the choice of action.

More generally, our results shed light on the necessity of distinguishing judgment and choice of action to better understand the factors that influence each of these two moral responses. They also point to the need for a more systematic analysis of the coherence between these two responses to dilemmas. Although, as Tassy et al. (2013) argue, these two aspects of moral evaluation are not necessarily related, it may be possible that the first response influences the second. In our study, judgment on the acceptability of the sacrificial action may have influenced the choice of action. For example, Capraro et al. (2019) have shown that asking participants “what they think is the right thing to do from a moral point of view” before making a decision can act as a “moral nudge,” increasing prosocial and altruistic behaviors. One solution would be to ask participants to make a choice before producing a moral judgment, as is the case, for example, in many studies that use sacrificial dilemmas as an experimental tool to study moral driving behavior (e.g., Bruno et al., 2022, 2023). However, here again, it is also possible for the second response to be influenced by the first, with participants producing a moral judgment consistent with their choice (e.g., “this outcome is more acceptable because it’s the one I chose”). Given the potential impact of question order, future research should explore this factor more systematically.

While the presence of a scenario increased the perceived plausibility of the sacrificial action, it had no effect on the measures of perceived realism. Indeed, participants perceived the dilemmas without a scenario to be just as realistic as the dilemmas with a scenario. This result is worth considering, particularly in light of other studies which, contrary to our results, show that providing additional information in the scenario increases the realism of the dilemmas (e.g., Carron et al., 2022; Körner and Deutsch, 2023). In these studies, however, the dilemmas were based on historical events (i.e., they had occurred in real life) whereas our dilemmas were purely hypothetical. Trolley-type dilemmas are unrealistic and sometimes absurd (Bauman et al., 2014) and, in our study, the addition of a scenario did not alter either the perceived realism or the participants’ feelings, or even their moral judgment.

More generally, these results raise the question of the value of providing factual and contextual information through the scenario for these kinds of dilemmas, at least when it comes to measuring individual differences in moral views and judgments. Scales composed of items similar to sacrificial dilemmas without a scenario might therefore be more appropriate for assessing moral judgment, if action is not of interest. Among these scales, the Oxford Utilitarianism Scale (OUS) (Kahane et al., 2018) has the advantage of probing utilitarianism in its two dimensions: the “negative” dimension, which corresponds to a permissive attitude towards instrumental harm (e.g., It is morally right to harm an innocent person if harming them is a necessary means to helping several other innocent people) and the “positive,” altruistic dimension, which refers to the concern to do good, even at the cost of self-sacrifice, knowing that the well-being of each individual is important (e.g., from a moral point of view, we should feel obliged to give one of our kidneys to a person with kidney failure). This scale, translated and validated in 15 languages (Erzi, 2019; Carron et al., 2023; Oshiro et al., 2024), is an alternative to classic dilemmas that not only focus solely on the negative dimension of utilitarianism, but are also highly criticized because they have not always been standardized: scenarios vary from one study to another and translations of the original English version have not always been validated. As Kahane et al. (2018) pointed out, the OUS is a measure of moral views and judgments, but not of behavior or intentions to act. For these behavioral measures, which are more sensitive to contextual information, sacrificial dilemmas seem to be more appropriate tools, if the information provided in the scenarios is taken into account.

While much work on emotions and moral judgments has focused on the effects of experimentally induced emotions (e.g., Brigaud and Blanc, 2021; Valdesolo and DeSteno, 2006), trait emotions (e.g., Chapman and Anderson, 2014; Choe and Min, 2011; Gleichgerrcht and Young, 2013) and emotional impairments (e.g., Koenigs et al., 2012; Patil and Silani, 2014) on individuals’ moral responses, little attention has been paid to the emotional state of individuals faced with classic sacrificial dilemmas, such as Greene et al. (2001). In our study, we have focused on emotion as a variable that changes throughout the formation of a moral evaluation. Our results indicate that emotional reactions were the same whether the scenario was present or absent. In both conditions, participants reported feeling more guilt after providing moral responses than at the beginning of the experiment (for similar results see Choe and Min, 2011; Horne and Powell, 2016). Miceli and Castelfranchi (2018) explain guilt as related to one’s sense of responsibility for a harmful attitude or behavior, and implies a negative moral self-evaluation. Participants also reported feeling more disgust after giving moral responses. Surprisingly, they also felt less anger. This result can be explained in the light of recent work which suggests that disgust and anger towards moral violations are in fact distinct in terms of the situations in which they are activated. Disgust (but not anger) is related to other-targeting violations whereas anger (but not disgust) is related to self-targeting violations (e.g., Bruno et al., 2023; Molho et al., 2017; Tybur et al., 2020). Probing participants’ specific emotions (e.g., anger and disgust; Haidt, 2003) when faced with sacrificial dilemmas may help to better understand the differences between the two moral responses (i.e., judgment and choice of action). Unfortunately, in our study, the correlations were too weak to conclude that there was a link between these emotions and each of the two moral responses.

## 5 Limitations and future directions

One limitation of our study is that participants identified emotions after responding to moral dilemmas (i.e., after deliberation). In particular, they reported feeling more guilt and more disgust after providing moral responses than at the beginning of the experiment, two emotions that are classically associated with moral violation (e.g., Mancini et al., 2022). These results do not provide information about the emotions people felt when they were confronted with the dilemmas and before they had to make decisions. Horne and Powell's (2016) results are therefore particularly enlightening on the effect of sacrificial dilemmas on individuals’ feelings insofar as they are among the few studies that have probed emotional feelings before and immediately after the presentation of moral dilemmas. They showed significant emotional change from pre-test to post-test indicating that reading a sacrificial moral dilemma led to increased negative emotions and decreased positive emotions. In summary, while the assessment of emotional states both before and after the experiment provides insights into overall emotional shifts, it may not fully capture the specific emotions evoked directly by the moral dilemmas. That is, the method herein used, although offering an account of the emotional experience throughout the overall experiment, might overlook the immediate emotional responses triggered by individual scenarios. Future studies could improve the precision of emotional measurements by assessing emotions immediately following each dilemma, as suggested in previous research (e.g., Choe and Min, 2011; Szekely and Miu, 2015; Horne and Powell, 2016).

Another limitation stems from the self-report measures, which require participants to be aware of their emotional state and able to report it accurately. Methods that are able to measure unconscious emotional experiences, such as Noldus’ FaceReader (Noldus, 2014), an automated facial coding software, would probably be more appropriate and reduce the potential risk of desirability bias. Further studies could measure unconscious emotional experiences before and after reading the dilemma to provide a better understanding of the moral decision-making process.

Regarding the type of dilemmas used in this study, the combination of impersonal dilemmas with non-personalized factual information in the scenario may have over favored the cognitive system of the dual-process theory (Greene, 2007; Greene et al., 2001, 2004, 2008) and reinforces the occurrence of the utilitarian action. Therefore, we recommend to extend this finding by considering personal dilemmas often known to elicit emotional responses. In the latter case, as long as the emotional system is rapidly activated, the factual information provided in the scenario should not have the same weight on moral responses.

As additional limitations, several aspects of the experimental design could be improved. First, while a 6-point Likert scale was used to capture more nuanced moral judgments, an alternative approach could have been to employ a dichotomous scale (e.g., yes/no). This simpler format might provide clearer distinctions between moral decisions, potentially facilitating a more straightforward interpretation of results. Second, the population sample used in the present study was limited in variability of age (younger, and first year university students) and gender. Other relevant factors, such as participants’ educational level, professional category, and socioeconomic status, were not measured. These variables could potentially influence moral judgments and offer a more comprehensive understanding of how individual differences may shape decision-making processes. All these limitations are avenues for improvement for future research, which will be tasked with pursuing this promising line of research.

The last but not the least line of research concerns ethical decision-making which is rarely an entirely solitary affair. Indeed, contextual information such as perceptions of others’ thoughts and opinions (i.e., “mind perception”) can also influence and interact with moral responses (e.g., Gray et al., 2012). Some studies have therefore investigated moral responses in group conversation situations (e.g., Smith et al., 2023) which represented a relevant context to newly investigate the power of the scenario.

## Data Availability

Datasets are available on request: The raw data supporting the conclusions of this article will be made available by the authors, without undue reservation.

## Footnotes

^1^Note that while the “Vitamins” dilemma is classified as a personal high-conflict sacrificial dilemma, it differs from the others in that it involves compromising an individual’s health rather than directly causing their death.

^2^ https://www.qualtrics.com
